# IgG1 and IgG4 antibodies against *Aedes aegypti* salivary proteins and risk for dengue infections

**DOI:** 10.1371/journal.pone.0208455

**Published:** 2019-01-02

**Authors:** Jenny C. Cardenas, Papa M. Drame, Karina A. Luque-Burgos, Juan D. Berrio, Elsi Entrena-Mutis, María U. González, Daisy J. Carvajal, Lady Y. Gutiérrez-Silva, Lucio D. Cardenas, Tonya M. Colpitts, Christopher N. Mores, Berlin Londono-Renteria

**Affiliations:** 1 Laboratorio Clínico, Hospital Local Los Patios, Norte de Santander, Colombia; 2 Department of Entomology, Kansas State University, Manhattan, Kansas, United States of America; 3 Laboratorio Clínico, Hospital Erasmo Meoz de Cúcuta, Norte de Santander, Colombia; 4 Grupo Investigaciones en Enfermedades Parasitarias e Infecciosas, Universidad de Pamplona, Pamplona, Norte de Santander, Colombia; 5 Laboratorio Clinico, E.S.E Hospital Emiro Quintero Cañizares, Ocaña, Norte de Santander, Colombia; 6 National Emerging Infectious Diseases Laboratories (NEIDL), Department of Microbiology, Boston University School of Medicine, Boston, MA, United States of America; 7 Department of Global Health, Milken Institute School of Public Health, The George Washington University, Washington DC, United States of America; Faculty of Science, Ain Shams University (ASU), EGYPT

## Abstract

Dengue virus (DENV) is an arbovirus responsible for a significant number of deaths in Latin America. This virus is transmitted through the bite of *Aedes aegypti*, the main mosquito vector, and *Ae*. *albopictus*. During blood uptake, the mosquito injects its saliva into the host to facilitate the feeding process. Mosquito saliva contains potent immunogens capable of inducing antibody production directly related to mosquito bite exposure intensity and disease risk. In this study, we first determined the DENV infection status by two different DENV non-structural protein 1 (NS1) based rapid tests and qRT-PCR, then measured the levels of IgG1 and IgG4 antibodies against salivary proteins of *Ae*. *aegypti* female mosquitoes in volunteers living in a dengue endemic area. Our results show that people with a positive DENV diagnosis present higher levels of IgG4 antibodies than people with a negative diagnostic test, and that these antibody levels were higher in people with secondary DENV infections. With this study, we show that detection of IgG4 antibodies against mosquito saliva may be a reliable method to evaluate the risk of dengue infection.

## Introduction

Dengue fever is an important disease in most tropical countries caused by dengue virus (DENV) [1, 2]. In Colombia, more than 80% of the territory is suitable for DENV transmission due to the presence of the main vectors, *Aedes aegypti*, and the recently introduced Ae. *albopictus* [2]. Although the majority of infections with DENV are asymptomatic, approximately 20% of infections result in a febrile illness, known as dengue fever (DF) characterized by headache, muscle pain and even rash among other symptoms [3, 4]. DF is often indistinguishable from other infectious diseases also endemic in the tropics including malaria and leptospirosis [5]. A small percentage of dengue fever cases progresses to the severe forms, classically known as dengue hemorrhagic fever (DHF) and dengue shock syndrome (DSS) [6]. These forms are characterized by an increase in vascular permeability, hypovolemia, and petechiae that may lead to death within 24 hours if not treated effectively [7]. However, early diagnosis seems to have a significant positive impact on patient recovery following appropriate treatment [8, 9].

Several factors have been associated with the geographical distribution of severe cases of dengue. One of these factors is the concurrent transmission of more than two DENV serotypes in a specific area [10, 11]. In Colombia, all four DENV serotypes (DENV 1–4) are currently circulating; therefore, there is a significant probability for severe disease due to the antibody-dependent enhancement (ADE) phenomena, where sub-neutralizing antibodies from a previous DENV infection may enhance a further infection with a different serotype [7, 12, 13]. A systematic review of dengue infection in Colombia shows severity rates for DENV infection ranging from 4.1% and 16.2% between 2005 and 2011 [14]. Although the mechanisms for DENV pathogenesis are still not well understood, research suggests that factors like age, genetic background, and dengue history may influence the disease’s outcome [15, 16]. Consequently, an early and accurate diagnosis, as well as an evaluation of disease history, is critical in determining patient management and treatment options [17, 18].

Public health agencies rely on both disease incidence and mosquito surveillance to measure disease transmission intensity and to develop the best control policies [19, 20]. As for most mosquito-borne diseases, dengue prevention also relies predominantly on vector control interventions [21]. Current entomological methods for disease risk measurement are laborious and often cannot pinpoint individual exposure [22, 23]. During transmission, mosquito saliva is deposited in the skin along with DENV [24]. This saliva contains potent immunogenic proteins able to induce antibody responses closely related to the intensity of exposure to mosquito bites and the risk of disease transmission [25]. Our previous studies have shown that antibodies against mosquito salivary proteins represent a useful tool to measure the degree of exposure to vector bites and to calculate the risk of disease transmission [25–27].

In humans, IgG antibodies are closely related to the intensity of exposure to mosquito bites with IgG4 as the main subclass associated with exposure to salivary allergens [28, 29]. IgG4 antibodies may interact with other antibody types modifying or decreasing their activity. Exposure to mosquito saliva also induces production of IgG1 antibodies and very low concentrations of IgG2 and IgG3 [28, 30, 31]. The purpose of this study was to measure antibodies against *Ae*. *aegypti* salivary gland extract (SGE) and to determine whether there was any association between the intensity of exposure to mosquito bites and the presence of primary versus secondary dengue infections in residents of endemic areas in Colombia with the aim of validating the use of such IgG antibodies against mosquito salivary proteins as a reliable marker for dengue infection risk.

## Materials and methods

### Ethical considerations

The protocols and methods for this study were reviewed and approved by the University of Pamplona and Los Patios Hospital and the Louisiana State University Ethics Review Board. The objectives of the research were clearly explained to each potential participant (guardian or parent for children) and written informed consent was obtained prior to sample collection.

### Study area

This study was conducted in two cities, Los Patios and Ocaña, of the State of Norte de Santander located in the northeast of Colombia. The State shares borders with Venezuela and is the principal area of commerce with Venezuela and the Caribbean; consequently, agriculture is one of the main sources of income. Norte de Santander is a sub-tropical area with an annual average rainfall of 1100 mm. There are two rainy seasons: March–June, and September-December. Consequently, DENV infections peak between mid-August and mid-October and between December and February [32]. Los Patios and Ocaña are ones of the most endemic cities for DENV in the country [14].

### Study population and sampling

Blood samples (5 mL each) were collected from all voluntary participants with less than five (5) days of symptoms seeking medical care at the Los Patios (San Juan de Dios Hospital) and Ocaña (E.S.E Hospital Emiro Quintero Cañizares). The cohort was set up in January 2013 and recruitment performed until September 2014. Each sampled blood was centrifuged, and the serum was isolated to perform DENV non-structural protein 1 (NS1)-based rapid test immediately. The remaining serum was stored at -20^o^ C until shipped to the United States. RNA was later extracted and used to detect the presence of viral genome by quantitative, reverse-transcriptase polymerase chain reaction (qRT-PCR).

### Dengue diagnostic tests

Both the Xerion DENGUE Ab IgG / IgM antibody and the Xerion DENGUE antigen (Ag) to detect the NS1 antigen (Xerion—IMEX group, Bogota) were used to determine DENV infection following factory recommendations. In brief, shortly after the collection of the human serum, the XERION DENGUE Cassette and dropper were carefully removed from the aluminum packaging and identified with the patient code. With the dropper upright, the serum sample was collected and transferred to the absorbent orifice of the Cassette (approximately 5μl serum). Then three (3) drops of buffer (~ 90μl) were added to the absorbent orifice of the Cassette preventing bubbles from forming. Results were recorded after 10 minutes of incubation. According to the test manufacturer, primary dengue infection is revealed by an IgM+/IgG- (between 1–7 days of symptoms) and an IgM+/IgG+ (between 1–7 days of symptoms) test, while a secondary infection is defined by an IgM-/IgG+ test anytime during the course of the symptoms. Molecular testing through qRT-PCR was also performed using the conditions and primers published elsewhere [25, 27, 33].

### Preparation of salivary gland extract (SGE) from *Aedes aegypti*

*Aedes aegypti* females (Rockefeller strain) 5 to 10 days old, were anesthetized with cold and then washed with 70% ethanol. For dissection, each mosquito was placed in a drop of 1X phosphate buffered saline (PBS), pH 7.2. Pairs of dissected salivary glands from 20 female mosquitoes were harvested in 1X PBS and allowed to freeze at -80° C and thaw four times to induce cell disruption and protein release. The resulting SGE was stored in aliquots at -80° C until use. Protein concentration was determined using Thermo Scientific NanoDrop (Thermo Fisher Scientific, Wilmington, DW).

### Evaluation of the level of antibodies against *Aedes aegypti* SGE

To test the level of antibodies against SGE, we used 96-well ELISA plates (Nunc Maxisorp, Nalgene Nunc International, Rochester, NY) coated with 100 μl / well of 0.5 μg / ml of *Ae*. *aegypti* SGE prepared in coating solution (Kierkegaard and Perry Laboratories, Gaithersburg, MD) and incubated overnight at 4° C. Plates were then blocked for 1 h with 1% milk powder in 1X PBS (blocking buffer) at 37° C and incubated with 100 μL / well of a 1/100 dilution of human patient sera in blocking buffer at 37° C for 2 h. The plates were washed three times with wash solution (1X PBS + 0.1% Tween 20) (Sigma–Aldrich, St. Louis, MO) and incubated with 100μL / well of horseradish peroxidase (HRP) labeled goat anti-human IgG4 (1: 1000), or IgG1 (1: 500) antibodies (Life Sciences, Grand Island, NY) at 37° C for 1 h. Colorimetric development was then obtained using 100 μL / well of One-Solution Microwell Tetramethyl Benzidine (TMB) substrate (Gene-Script, Piscataway, NJ) and incubated for 15 min at room temperature. The reaction was stopped with 100μL / well of stop solution (1M phosphoric acid), and the absorbance was measured at 450nm. Each sample was tested in duplicate, and three controls were included in each plate: 1) blank control: two sources without SGE for control for non-specific color induction for any of the reagents used in the assay; 2) negative control: two wells with SGE but no human serum to control for any non-specific color induction of the coating antigen; and 3) positive control selected from previous anti-mosquito saliva Ab testing study in Colombia for the control of plate to plate variations and to normalize the optical density (OD) values.

### Statistical analysis

The difference between two independent groups (i.e., antibody levels between dengue positive and dengue negative subjects) was determined using the Mann-Whitney test with a p-value <0.05. Comparison of more than three groups was tested with the Kruskal-Wallis test. Correlation between to independent parameters was done using Spearman correlation method. Statistical analysis was performed using GraphPad Prism, version 7 (GraphPad Software Inc., La Jolla, CA).

## Results and discussion

Our findings suggest that individuals with positive dengue test and secondary infections presented significantly higher IgG4 antibodies against mosquito saliva compared to individuals with negative dengue test and with primary dengue infections, respectively. This pilot study represents the first approximation to compare mosquito bite exposure levels in people with primary versus secondary DENV infections describing an association between history of disease and level of exposure to vector bites.

### Dengue infection history

**Fig 1:**
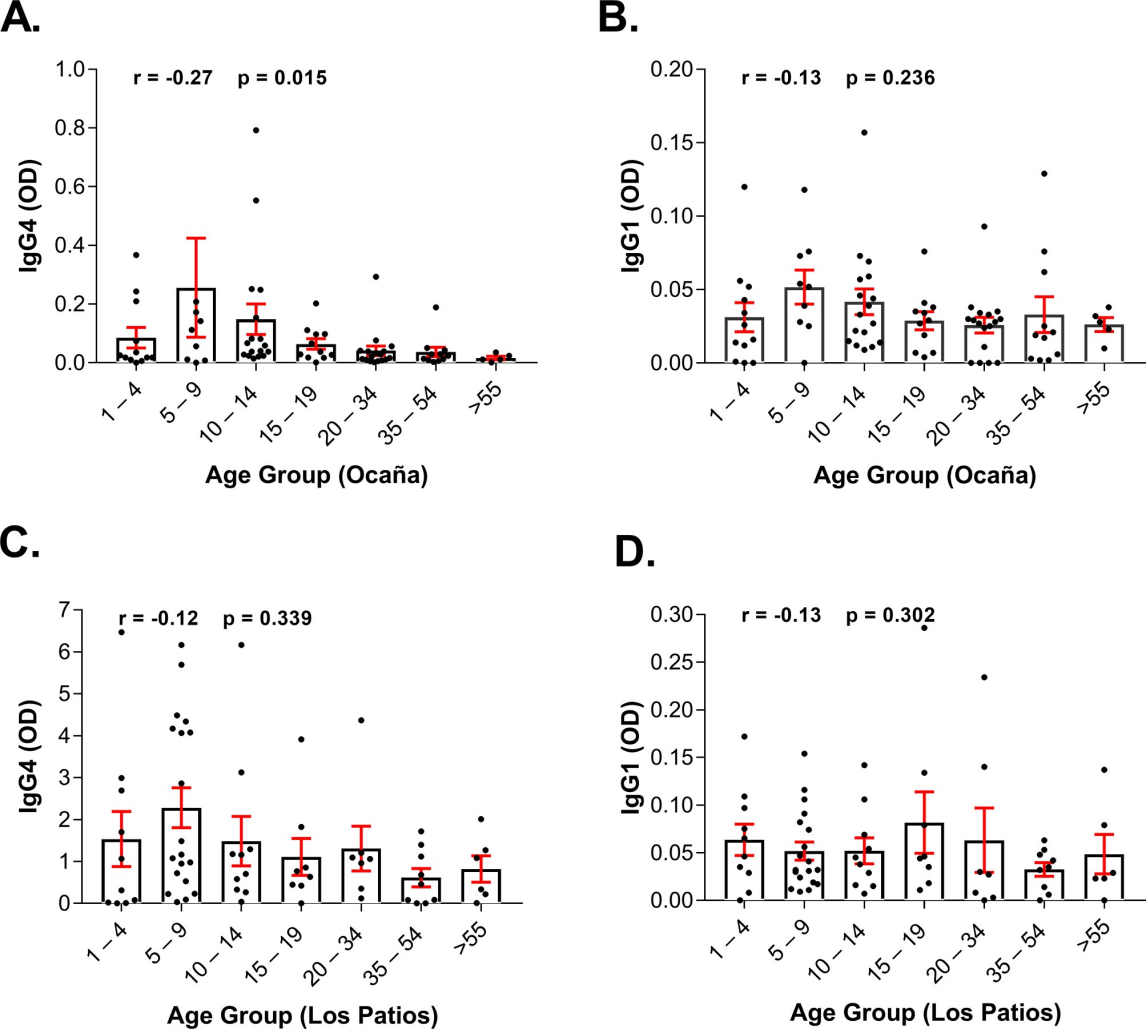
IgG4 and IgG1 antibody responses to *Aedes aegypti* salivary gland extracts according to age in Los Patios and Ocaña. The distribution according to age of IgG4 and IgG1 specific to *Aedes aegypti* saliva is plotted for Los Patios (A and B, respectively) and for Ocaña (C and D, respectively). Individual responses are represented by the dark dots and boxes represent means of group individual antibody responses with their standard errors (vertical red bars). “r” and “p” values were obtained from the non-parametric Spearman correlation method.

A total of 151 participants were included in this study. The mean age in our study was 21. 3 years old (from 1 to 92 years old). There were no significant differences in the mean age between the two study sites. Specifically, mean age in Los patios was 20.57, and in Ocana, the mean age was 22.06 (p = 0.0635). The levels of IgG4 and IgG1 specific to *Aedes aegypti* saliva according to age in the two studied sites (Ocaña and Los Patios) are represented in Fig 1, and the number of tests performed on the study sample and their infection status according to age in the whole studied population is described in **Table 1**. In Colombia, most dengue cases are presented for children ages between 4 to 14 years old, and severe cases in the area are mainly reported in this age group [34]. Accordingly, our results show higher levels of anti-mosquito saliva IgG4 and IgG1 antibodies in Ocaña and higher levels of specific IgG4 in Los Patios in the groups 1–4 years old, 5–9 years old, and in the 10–14 years old group compared to older age groups. In addition, the prevalence of DENV assessed by qRT-PCR in the total studied population is higher in these three age groups.

**Table 1:** Distribution by age of samples positive for dengue tests performed in the study.

Age group (Years old)	n	NS1-IgG	NS1-IgM	NS1-Ag	qRT-PCR
1–4	22	66% (10/15)	13% (2/15)	79% (15/19)	50% (11/22)
5–9	28	64% (9/14)	7% (1/14)	39% (7/18)	60% (17/28)
10–14	27	60% (12/20)	10% (2/20)	54% (13/24)	44% (12/27)
15–19	19	76% (10/13)	0% (0/13)	74% (14/19)	26% (5/19)
20–34	24	52% (11/21)	19% (4/21)	54% (12/22)	42% (10/24)
35–54	20	64% (11/17)	18% (3/17)	41% (7/17)	35% (7/20)
>55	11	85% (6/7)	0% (0/7)	36% (4/11)	36% (4/11)
**Total**	**151**	**64% (69/107)**	**11% (12/107)**	**55% (72/130)**	**44% (66/151)**

Circulation of more than two serotypes in a specific area is a risk factor for severe dengue. We found by qRT-PCR that all four DENV serotypes are represented, with DENV 2 as the main serotype in single (45%: 20 out of 44) as well as mixed infections (90%: 9 out of 10).

In our study, 55% (72 out of 130) of the total population presented NS1-Ag. That prevalence was 52% (28 out of 54) in Los Patios and 58% (44 out of 76) in Ocaña (Table 2). In addition, 64% (69 out of 107) of samples presented NS1-IgG positive results suggesting a higher prevalence of secondary infections. That prevalence was 53% in Ocaña and 56% in Los Patios (**Table 3**). This is consistent with previous studies showing secondary infections prevalence in Colombia between 50% and 68% [34, 35]. Severe dengue is usually observed in patients with secondary infection caused by a serotype different from the one responsible for the primary infection [7, 36]. Detecting secondary infections early in the illness is an important factor not only to predict prognosis but also to select treatment and monitoring options [36, 37]. Previous studies reported that the NS1-IgG antibodies first appear after day 14 in primary infections, but its concentration is significantly incremented in secondary infections where it can be found from day 2 [38, 39].

**Table 2:** Results of the NS1 Ag-based rapid tests in Ocaña and in Los Patios.

City	NS1-Ag (+)	NS1-Ag (-)	Total
Los Patios	28	26	54
Ocaña	44	32	76
**Total**	72	58	130

**Table 3:** Results of the NS1 IgG/IgM-based rapid tests in Ocaña and in Los Patios.

	Ocaña				Los Patios			Grand total
	NS1-IgG (+)	NS1-IgG (-)	Total		NS1-IgG (+)	NS1-IgG (-)	Total	
NS1-IgM (+)	1	1	2	NS1-IgM (+)	10	0	10	12
NS1-IgM (-)	44	36	80	NS1-IgM (-)	14	1	15	95
**Total**	45	37	82	Total	24	1	25	107

### Exposure to salivary antigens and dengue history

In areas suitable for vector-borne disease transmission, people are more often bitten by uninfected than infected mosquitoes and if anti-salivary proteins antibody levels depend on exposure to mosquito bites [25] it is very likely that the presence of antibodies against mosquito saliva may have an impact on disease progression, especially since several salivary proteins are important for DENV replication [40]. In the current study, we found a negative correlation between age and SGE-IgG4 antibody levels in both study sites, although the relation was only significant in Ocaña (r = -0.27, p = 0.015) (Fig 1). The relationship between age and IgG4 antibody levels is still not well understood. Previous studies reported a negative correlation between age and IgG4 antibodies, a decrease of IgG4 antibodies with age [31, 41] suggesting the development of immune-related tolerance thus higher levels of IgG4 are found in younger children with a decrease or even a plateau in individuals 20 years or older [41, 42]. This could also mean a higher exposure to mosquito bites in younger children.

**Fig 2:**
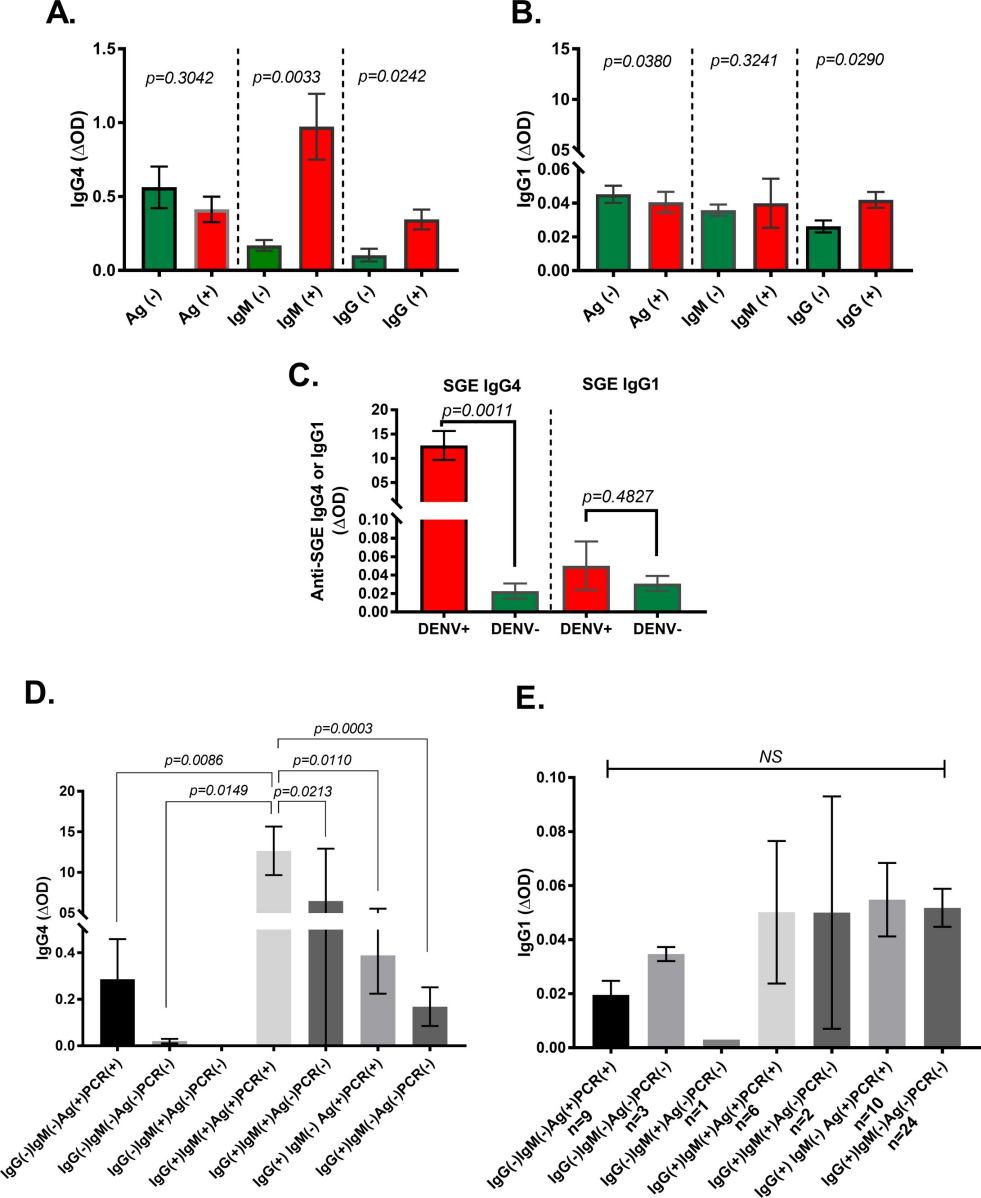
Representation of the IgG1 and IgG4 antibody levels by dengue test results. Anti-SGE IgG4 (A) and IgG1 (B) antibody levels by rapid NS1 test results are represented. C. represents the anti-SGE IgG1 and IgG4 antibody levels using concordant NS1-IgG/IgM and NS1-Ag test results. D and E describe the IgG4 and IgG1 antibody levels in groups determined using all test results with concordant NS1-Ag and RT-PCR. Boxes represent the means of group individual antibody responses along with their standard error (vertical bars).

In general, SGE-IgG1 tend to appear first early in the exposure to mosquito bites, the longer the exposure, the antibody responses switch from SGE-IgG1 and IgM to SGE-IgG4 antibodies, suggesting SGE-IgG4 are more correlated to chronic exposure to mosquito bites. Previous studies indicated that the genetic makeup and behavioral preferences might impact the SGE-IgG4 antibody response profile [43]. When comparing the level of antibodies according to the NS1-IgG/IgM results, we found that SGE-IgG4 antibody levels were significantly higher in people with a positive result than the other groups (Fig 2A). We did not see any significant differences in the level of SGE-IgG1 antibodies among groups (Fig 2B). Analysis of individuals with dengue test concordant results revealed that anti-saliva IgG4 antibodies are higher in people with a positive NS1-IgG/IgM and qRT-PCR results compared to the ones with negative NS1 IgM/IgM and qRT-PCR results (Fig 2C). This is concordant with previous assumptions that higher exposure to mosquito bites correlate with higher risk of suffering disease [25, 44]. A such difference was not observed with SGE-IgG1 antibodies (Fig 2C). In allergic responses, high IgG4 antibody levels are usually associated with protective anti-inflammatory responses by preventing IgE-mediated mast cell activation [28]. Salivary antigens induce significant infiltration of dengue target cells to the bite site [45]. Thus, higher SGE-IgG4 may result in less inflammatory reactions that can be translated into less substrate for the virus to replicate.

A recent study shows that antibody-mediated DENV pathogenesis and disruption of endothelial barrier leading to a possible blood leakage is exacerbated by mosquito saliva in patients with cross-reactive enhancing antibodies often present during secondary infections [46], while other studies suggest that an increase in SGE antibodies may be detrimental in dengue infections. Previous studies suggest that immunization with a D7 protein from *Culex spp*. mosquitoes enhances severity in West Nile virus infection [47]. Results of these two aforementioned studies are in concordance with our recent studies showing that the *Ae*. *aegypti* D7 salivary protein can inhibit DENV replication *in vivo* and *in vitro* [48] and antibodies against it may block this inhibitory property leading to higher virus replication [49]. Since higher SGE-IgG4 was found in secondary infections and although, only a fraction of those secondary infections progress to severe disease, characterizing the immune profile could lead to a better understanding of the role of anti-salivary antibodies in dengue pathogeneses.

The current study has some limitations. The first is that the sampling method used does not allow for testing of causal relationships. More field studies are needed to characterize the IgG antibody subclass profiles against salivary proteins in people with severe and non-severe disease. The second limitation of the study is that we have used a whole salivary extract of *Ae*. *aegypti* and some of the molecules found immunogenic may share epitopes with the saliva of other hematophagous arthropods such as both *Anopheles* and *Culex* genera that are also present in the studied areas. Therefore, we cannot exclude that the levels of antibody assessed here are party explained by the exposure to these non-*Aedes* vectors.

## Conclusion

Mosquito saliva plays a significant role in the transmission of arboviruses like DENV. People living in dengue-endemic areas in the tropics are exposed continuously to bites favoring the production of IgG4 antibodies against mosquito saliva. These antibodies are closely related with both the level human-vector contact and to dengue history. This study represents the first approximation to evaluate the relationship between exposure to vector bites and presentation of dengue infection including the IgG1 and IgG4 antibodies against dengue primary and secondary infections. However, further studies are needed to characterize a marker for risk of secondary infections. Our current study is limited to describe the behavior of such antibodies in the presence of primary/secondary infection by rapid tests, however, immunological and molecular methods may give a better a proximation to determine the role of anti-salivary antibodies in pathogen transmission and disease severity.

## Supporting information

S1 File Row data that were used to generate the graphs presented in this paper. (XLSX)
